# Drug allergies in primary care practice in Romania: a questionnaire - based survey

**DOI:** 10.1186/1710-1492-10-16

**Published:** 2014-04-01

**Authors:** Polliana Mihaela Leru

**Affiliations:** 1 Carol Davila University of Medicine and Pharmacy Bucharest, Bucharest, Romania; 2 Allergology Department, Colentina Clinical Hospital, Av. Stefan cel Mare no.19-21, District 2, 020125 Bucharest, Romania

**Keywords:** Drug allergies, Education, Primary care

## Abstract

**Introduction:**

Recent data from literature have shown many difficulties in managing allergic diseases in primary care in most countries and a consequently clear need for standardized educational programmes. Drug allergies represent an important medical issue for general practitioners (GPs) in Romania, though no national data about incidence, severity and management exist.The aim of our study was to evaluate epidemiological aspects of drug allergies in primary care practice in Bucharest, especially the diagnostic and therapeutic attitudes of family doctors and their need for education and training in this field of pathology.

**Findings:**

A questionnaire with 21specific questions was addressed to 800 family doctors from Bucharest, either directly or via internet, with a response rate of 31,87%.

The answers showed a significant interest of GPs in drug allergies, which are considered an increasing pathology. Almost half of the responders had never attended any form of education in allergology and 96% expressed a clear interest to participate in specialized educational programmes. We have noticed an underestimation of the severity of drug allergy, a surprisingly high percentage of allergy skin tests or blood tests recommended by GPs without specialist advice, and persistant confidence in alternative medicine.

**Conclusions:**

We concluded that the attitude towards and the competence regarding drug allergies of GPs in this study, as well as their collaboration with allergists, are not standardized and updated according to current guidelines. Further educational programs for GPs in drug allergies, based on standardized guidelines and national epidemiological studies for evaluation of drug allergy-related morbidity and mortality are needed.

## Background

Drug hypersensitivity reactions represent an important medical issue associated with significant morbidity and mortality, with still unknown incidence in many countries [1]. Large-scale epidemiological studies are missing and confusion between non-immunological drug reactions and drug allergies is frequent in clinical practice [2]. Previous studies reported a large number of medication errors and hospital admissions due to adverse drug events in outpatient settings [3]. There is no national registry of drug hypersensitivity reactions in Romania and no clear data about medical attitudes of general practitioners (GPs) in this field. The limited number of allergists and the reduced time of specialized training in allergology and clinical immunology during university studies induce many difficulties in managing allergic diseases in general and drug allergies in particular. The position paper on allergy management in primary care, recently published in *Allergy*, highlighted the need for sustained and homogenous education programmes and large access of GPs to training, mainly for the most prevalent and potentially severe allergic diseases [4].

## Findings

The aim of the study was to evaluate epidemiological aspects of drug allergies in primary care practice in the biggest city of Romania, as well as the family doctors’ general opinion regarding this pathology, their diagnostic and therapeutic means of choice. The second aim was to evaluate the educational needs of GPs in the field of drug allergies, in order to organize further programs at a national level.

## Methods

The study was based on a questionnaire with 21 specific questions, addressed to 800 out of the total of 1400 family doctors from Bucharest, with available addresses, representing 57, 14% (Questionnaire annexed as Additional file 1). The questionnaire was administered either directly to doctors at their workplace, or by internet mailing via a specialized site over a period of three months, between March-May 2013. We obtained a number of 255 completed questionnaires, representing an overall response rate of 31, 87%, significantly higher via direct administration (76, 97%) than through internet. The gender and age distribution of responders was 187 women (73, 33%) and 68 men (26, 67%), with a mean age of 42 years. More than 90% of family doctors worked in their individual private offices and the rest in hospitals and private clinics. The majority of responders reported medical experience as family practitioners longer than 15 years.

## Results

Referring to the most frequent five diseases in adult patients encountered in their current practice, 77% of responders ranked the cardiovascular diseases first, followed by metabolic disorders- 46%, cancers – 46%, degenerative bone disorders and chronic respiratory diseases. Responses to the same question for the pediatric population showed a surprising predominance of immunological disorders (with 75% of responders), followed by infections, neuropsychiatric disorders, growth and digestive disorders. With regard to the importance of drug allergies in their medical practice, almost all answered that this pathology is very important (72%) or important (27%). The reported incidence of drug allergies was at least once a week for 27% of the GPs and at least once a month for 30%.The majority of respondents - 96% expressed their interest to participate in an organized form of training for drug allergies in the future.

Most of the respondents listed cutaneous rash, pruritus and facial edema as the dominant clinical picture, followed by respiratory symptoms, malaise and blood pressure changes. An important percentage of GPs (44%) answered that less than 10% of patients suspected to have drug allergies received allergists’ consultation and investigations, despite the fact that collaboration with specialized allergists is considered good by 71% of GPs, and not satisfactory by the rest of 29%. Most of the doctors – 83% consider that the incidence of drug allergies is on the increase in our country and 15% appreciate it as being stable. Incidence of drug allergies induced by new therapies among Romanian patients, compared to other countries, is considered higher by 33% respondents, similar by 26%, while 39% did not know how these compared. When asked what would be the responsibilities of general practitioners to the patients with drug allergies, 81% ranked immediate treatment as the first, and 72% referral to specialists, while about half of responders considered their main task to be monitoring, reporting, recording and counseling of treatment. In terms of therapeutic intervention, 91% of GPs currently recommend oral antihistamines and 64% oral corticosteroids, while 15% use alternative therapeutic methods. Alternative medicine, like acupuncture and homeopathy, were considered very efficient or efficient as diagnostic or therapeutic tools in drug allergy by 40% of respondents, not efficient by 12%, while 48% did not have any opinion on this subject.

## Discussion

**Figure 1:**
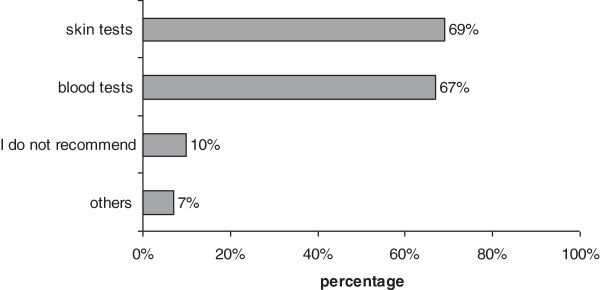
Reported GPs active diagnostic intervention in drug allergies.

**Figure 2:**
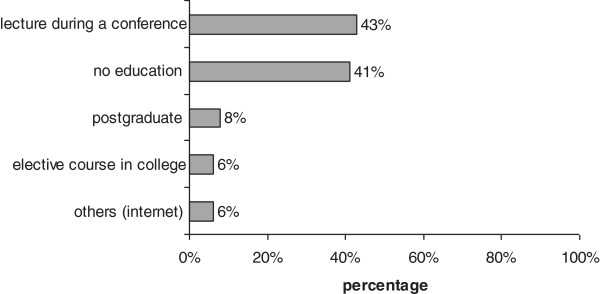
Reported education or training in drug allergies.

The overall response rate of family doctors to our questionnaire was good, showing their significant interest in drug allergies pathology. Most of them considered the topic to be very important or important and appreciated an increasing incidence of drug allergies in our country. The clinical diagnosis of drug allergy in primary care relies mostly on cutaneous signs,like rash and pruritus, while the systemic manifestations and severe forms appear to be underestimated. This aspect might be generally explained by the presentation of patients with moderate and severe drug allergies directly to hospitals, without GP consultation. A rather surprising answer revealed that 67% of GPs prescribe themselves allergy blood tests and 69% prescribe skin tests (Figure 1), despite declared good collaboration with specialists by 71% respondents. This aspect may indicate not standardized collaboration between GPs and allergists. Many GPs still rely, in their diagnostic and therapeutic approach to drug allergies on alternative methods, like homeopathy and acupuncture. Since 41% of family doctors have never attended any form of university or postgraduated education in allergic diseases and 43% have only attended lectures during conferences (Figure 2), 96% of respondents have expressed their interest for future training in this field of pathology.

Limitations of the study are mainly due to lacking national reference data, to differences between the Romanian health system and medical education by comparison to the ones of other countries and to the fact that the GPs who answered the questionnaire were the ones already interested in the topic.

The conclusion of the study is that the attitude and competence of GPs towards the issue of drug allergies, as well as their collaboration with allergists, are not standardized and updated according to current guidelines. Further evaluation of drug allergies management in primary care is needed. Updated training of family doctors in drug allergies is very important for improving the management and for reducing hospital burden and costs related to these diseases. Coordinated and sustained educational national programmes are needed, based on currently accepted international scientific and clinical standards in allergic diseases [5,6].

## Competing interests

The author declares no conflict of interest in relation with this paper.

## Supplementary Material

Additional file 1 Questionnaire. Click here for file
