# Supplementary material for: Drug allergies in primary care practice in Romania: a questionnaire - based survey
Source: Allergy Asthma Clin Immunol. 2014 Apr 1;10(1):16. doi: 10.1186/1710-1492-10-16 (PMC4021609; doi:10.1186/1710-1492-10-16)
Supplement: Additional file 1 — Questionnaire. [file 1710-1492-10-16-S1.doc]

**Drug Allergies in Primary care practice in Romania**

**A questionnaire –based survey**

Dear colleague

With this questionnaire we kindly ask you to express your opinion regarding drug allergies in your current medical practice . Your answers are very important for evaluation of this pathology in primary care practice and is aimed to contribute to identification of educational and medical assistance needs in this field .The information obtained will be considered strictly confidential and used only for scientific purposes . This activity is coordinated by the Department of Family Medicine from “Carol Davila “ University of Medicine and Pharmacy and College of Doctors from Bucharest .

Thanks for your collaboration !

1. General informations :

Number of registered patients :

Age: gender: duration of medical practice years.

Place of work : urban rural

1. What are the five most frequent chronic diseases that you encounter in your practice?

Adults:

Cardiovascular diseases metabolic diseases neoplasm

Degenerative joint pains chronic respiratory diseases

Children:

Growth and development disorders digestive diseases

Neuropsychiatric diseases infections immunological disorders

3. How do you appreciate the clinical importance of drug allergies in your daily practice?

Very important important less important

4. How often do you meet patients with drug allergies in your current medicine practice?

daily weekly 1-2 times per month 2-3 months hardly ever

5. How many patients with drug allergies you have in your evidence?

<10 20-30 30-50 50-100 >100 several hundred

I do not know

6. Have you participated in any form of training or education on drug allergies?

Elective course in college postgraduate lecture during a conference

Others no

7. Are you interested in participating in an organized form of training in the field of drug allergies pathology?

yes no

8. Which do you consider to be the most evocative signs and symptoms of drug allergies?

Cutaneous rash pruritus i facial edema malaise

respiratory symptoms high or low blood pressure others

9.What percentage of patients to whom you have suspected drug allergies received specialist consultations and investigations?

<10% 20% - 30% 50% >50%

10. What is the first recommendation you make to a patient with suspected drug allergy?

11. How do you appreciate the incidence of drug allergies in our country?

Stable in icreasing decreasing

12. What are the possible factors responsible for increased prevalence of drug allergies?

Increased drugs use individual risk factors genetic factors

Nutrition pollution stress others

13.What do you consider to be the most important classes of drugs responsible for allergic reactions in our country?

Antibiotics cardiovascular medication antidiabetic

Antiinflamatory vaccines contrasts agent cytostatic

Neurological and psychiatric medication vitamins others

14. How do you appreciate the patient exposure to novel drug allergies in our country compared to other countries?

equal higher lower I do not know

15. What do you think to be the main risks and consequences of drug allergy?

death chronic disease complication

therapeutic mistake psychiatric disorders impaired quality of life

social consequences others

16. At what percentage do you estimate the risk of death caused by drug allergies?

1- 2 % 3-5% 5-10% >10% >20% I do not know

17. What is the responsibility of the family doctor to the patient with allergies?

Immediate treatment referral to specialist counseling

Monitoring of treatment recording and reporting

18. Are you satisfied with the collaboration with specialists allergists and specialized assistance in your city?

Yes no

19. What investigations do you recommended to patients who probable drug allergies?

Skins tests blood tests others I do not recommend

20. What are the most recommended classes of medication used to treat drug allergies ?

Oral corticosteroids antihistamines immunomodulators

Alternative methods others

21. How do you appreciate the effectiveness of alternative methods like acupuncture and homeopathy in diagnosing and treating drug allergies?

Very Effective effective ineffective I do not know

| DOCTOR NAME |
| --- |
| SPECIALITY |
| PROFFESIONAL DEGREE |
| MEDICAL UNIT |
| CITY |
| PHONE EMAIL |
